# Questions About Aging and Later Life on Quora

**DOI:** 10.1093/geront/gnae060

**Published:** 2024-05-30

**Authors:** Reuben Ng, Nicole Indran

**Affiliations:** Lee Kuan Yew School of Public Policy, National University of Singapore, Singapore, Singapore; Lloyd’s Register Foundation Institute for the Public Understanding of Risk, National University of Singapore, Singapore, Singapore; Lee Kuan Yew School of Public Policy, National University of Singapore, Singapore, Singapore

**Keywords:** Concerns about aging, Public gerontology, Public opinion on aging, Text as data

## Abstract

**Background and Objectives:**

Gerontologists have yet to explore the types of questions individuals have about later life. Analyzing questions offers a unique perspective on how individuals make sense of age-related issues. Specifically, questions require people to articulate specific inquiries or doubts, thus providing an unfiltered glimpse into the public’s concerns and priorities vis-à-vis aging. We conduct a content analysis of questions posted on Quora that pertain to later life.

**Research Design and Methods:**

We compiled 2,950 questions posted across four topics on Quora: “Aging,” “Senior Citizens,” “Elders” and “Older People.” After applying our exclusion criteria, 658 questions were left for content analysis. These questions received more than 250 million views. Both deductive and inductive approaches guided the qualitative analysis.

**Results:**

Five themes emerged from the analysis. The biggest theme (30%; *N* = 195) dealt with ‘Practical Concerns’ (Theme 1). The next biggest theme (29%; *N* = 191) was about “Health and Well-Being” (Theme 2). Theme 3 was about the ‘Prolongation of Youth’ (16%; *N* = 110) and Theme 4 was about the ‘Science of Aging’ (15%; *N* = 97). Theme 5 covered ‘Existential Concerns’ (10%; *N* = 65).

**Discussion and Implications:**

There is a need to address concerns that the public has about aging, particularly those involving practical issues and health. Growing old is unavoidable and with the population aging at a rapid pace, assuaging such concerns is of paramount importance. By doing so, individuals can approach the aging process with greater clarity and an elevated sense of empowerment.

Analyzing inquiries related to aging and later life is one way to identify the attitudes, beliefs, and concerns of the public with regard to aging. This study aims to dissect such inquiries by conducting a content analysis of questions posted on Quora vis-à-vis old age. By undertaking this study, we aim to shed light on key age-related issues that captivate public imagination, uncover gaps in the knowledge of lay persons, and pinpoint areas in need of targeted interventions.

To date, no studies have looked at the types of questions individuals have regarding later life using a question-and-answer website. Past literature on public evaluations of old age analyzed content on social media platforms such as Twitter (Jimenez‐Sotomayor et al., 2020; Ng et al., 2022; Sipocz et al., 2021), TikTok (Ng and Indran, 2022), and Facebook (B. R. Levy et al., 2014), or relied on traditional methods such as interviews or surveys. Analyzing questions offers a unique perspective on how individuals make sense of age-related issues. In particular, questions require people to articulate specific inquiries or doubts, which could reflect concerns that may be more personal. Moreover, unlike interviews and surveys which elicit specific responses, questions on question-and-answer websites are presumably asked organically, thus providing an unfiltered glimpse into the public’s priorities and concerns. This will allow for the development of interventions aimed at addressing the needs of not just those in their later years, but all who will eventually undergo the aging process.

Founded in 2009, Quora is a question-and-answer website for sharing and acquiring knowledge on an array of topics. Users of Quora can post questions to seek insights or opinions from the wider community, and other users can then respond with answers based on their expertise, experiences, or viewpoints. Each question can receive multiple answers, and users can upvote or downvote both questions and answers. Users can also engage in discussions by leaving comments on answers or by following specific topics to stay updated.

Our reasons for selecting Quora as a platform for analysis were twofold. First, Quora has established itself as the leading question-and-answer website. Its massive user base—as of March 2023, more than 300 million unique users visit Quora every month (Ruby, 2023)—provides a vast pool of data for exploring attitudes, beliefs, and concerns about growing older. Other well-known question-and-answer websites like Answers.com and Yahoo! Answers have gradually waned in popularity, with the latter ceasing operations in 2021 (Glaser, 2018). Second, younger people make-up the bulk of Quora’s user base. Approximately 30% of users are between 25 and 34 years old, and 28% are between the ages of 18 and 24 (Ruby, 2023). This renders the site valuable for understanding the perspectives held by individuals before they transition to later life or as they care for their aging relatives.

## Literature Review

Aging is a natural phase of life. However, the meanings ascribed to it are highly subjective, making attitudes toward growing older diverse and complex (Ayalon and Tesch-Römer, 2018; Westerhof et al., 2014). Both positive and negative views of later life exist. On the positive side, later life is often celebrated as a time of fulfillment (Dudley et al., 2020; Wang, 2013), increased well-being (Blanchflower, 2021), and enhanced wisdom (Dionigi, 2015). Retirement, specifically, is widely embraced as a time of joy and meaning, an idea encapsulated by the expression “golden years” (Carr, 2019). Furthermore, advances in medicine have led to the coinage of expressions such as “60 is the new 40,” which reflect the notion that older adults today are living healthier and more active lives (Diehl et al., 2020).

On the negative side, later life is often thought to be taken an inevitable decline in both cognitive and physical abilities as well as a loss of independence (Lindland et al., 2015; Sweetland et al., 2017). Moreover, the current economic climate poses significant challenges for older people, necessitating them to work for a longer period (Lindland et al., 2015; Sweetland et al., 2017). This is compounded by the fact that the geographical dispersion of families may hinder the provision of direct social support to older adults (Lindland et al., 2015; Sweetland et al., 2017). Another misapprehension related to old age stems from the belief that social isolation is a common feature of later life (Köttl et al., 2022; Lindland et al., 2015; Morgan et al., 2021). Additionally, the increase in leisure time during retirement is commonly viewed more as a curse than a blessing (Bordia et al., 2020).

Unfortunately, there is a general consensus among scholars that negative stereotypes of old age abound (Dionigi, 2015; Ng et al., 2023) and that youth is adulated (Krekula et al., 2018). In fact, it is broadly acknowledged that there is a pervasive sense of anxiety surrounding the idea of aging. Lasher and Faulkender (1993) defined anxiety about aging as the anticipation of losses centered around the aging process. They conceptualized this anxiety as having four distinct dimensions: physical, psychological, social, and transpersonal. The physical dimension encompasses factors such as perceptions of one’s health status, concerns about physical self-efficacy, and the physical changes associated with aging. The psychological dimension includes factors such as perceived control, self-esteem, life satisfaction, and psychological disorders. The social dimension includes living conditions, perceived social support, as well as social and economic losses. The transpersonal dimension involves coping with mortality, searching for meaning in past and present life events as well as divinity.

According to Lasher and Faulkender (1993), these four dimensions of anxiety about aging are expressed in three specific fears: fear of aging, fear of being old, and fear of older people. The fear of aging is defined as the fear that arises from one’s personal aging journey. The fear of being old is rooted in the state of being old rather than the process of aging itself. The fear of older people refers to the sense of unease that comes with interacting with older adults. Evidence indicates that higher levels of anxiety about aging are correlated with ageist attitudes (Allan et al., 2014; Bodner et al., 2015).

The crux of Levy’s (2009b) theory of stereotype embodiment is that age stereotypes are internalized throughout one’s life span, eventually becoming self-definitions that predict health and well-being in later life. According to this theory, the assimilation of negative age stereotypes is linked to adverse health outcomes such as a higher risk of depression (Choi et al., 2022) and a lower sense of self-efficacy (Eibach et al., 2010; B. Levy, 2009b). On the contrary, positive age stereotypes are associated with improved functional health and well-being (B. Levy, 2009b; Söllner et al., 2022). There is also evidence that individuals who endorse negative age stereotypes in their younger years are more likely to experience a cardiovascular event in later life as compared with those who hold positive age stereotypes (B. Levy, 2009a).

Our qualitative content analysis is anchored in the following research questions: What concerns or misapprehensions do individuals harbor regarding later life? What aspects of aging are individuals seeking information on? What do the questions asked by individuals on Quora reveal about how old age is made sense of?

## Method

### Data set

We first created a new Quora account as navigating the site without an account limits the number of posts displayed. We then compiled a list of topics related to older adults and later life. Topics are a way of categorizing content on Quora. Users can follow specific topics to browse through questions and answers related to those topics in their feeds. Since no gerontological studies have been conducted on Quora, the search terms queried were adapted from past content analyses conducted on other social media platforms (B. R. Levy et al., 2014; Jimenez‐Sotomayor et al., 2020). The topics chosen were: (1) Aging, (2) Senior Citizens, (3) Elders, (4) Older People. Other commonly used terms such as “older adults,” “older individuals,” and “older persons” did not exist as topics on Quora.

**Figure 1.**
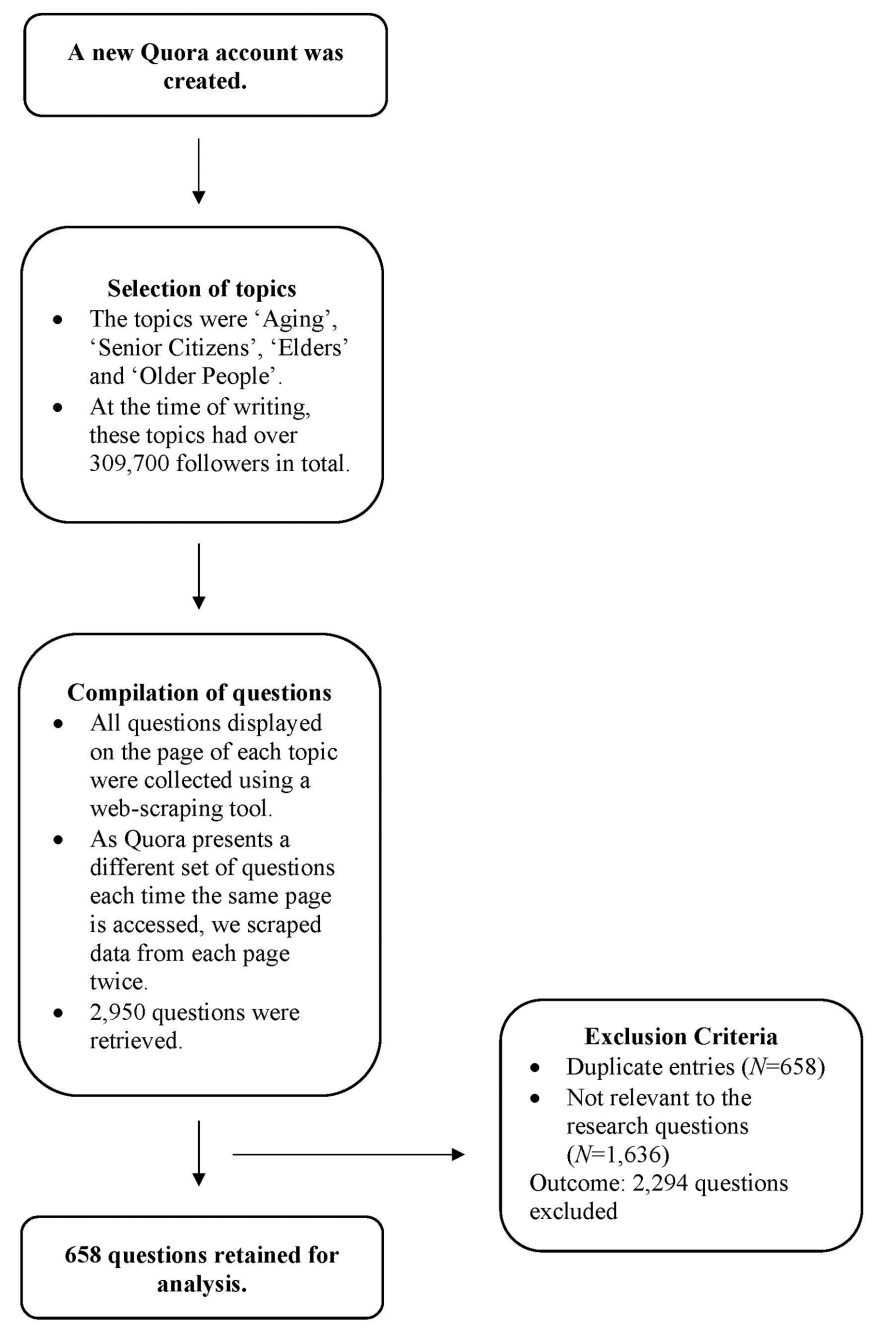
Process of compiling questions about later life posted by Quora users.

All questions displayed on the page of each topic were collected using Octoparse, a web-scraping tool. As Quora presents a different set of questions each time the same page is accessed, we scraped data from each page twice to build a more comprehensive data set. In total, 2,950 questions were retrieved. Next, we removed questions that were either duplicate entries (*N* = 658) or that were irrelevant to the study (*N* = 1,636). Examples of questions that were irrelevant include “What is the best age to teach a child how to swim?,” “How can I save money while buying from Amazon?,” and “Does getting older change our perception of Godzilla as an iconic monster figure?” Quora employs an algorithm to sort questions into topics (Li, 2020). As with any automated process, some degree of error is inevitable, leading to the possibility of certain questions being miscategorized. A total of 658 questions were left for content analysis. These questions amassed 253,524,750 views collectively and were posted between February 27, 2010 and February 5, 2023. The data collection process is shown in Figure 1.

### Content Coding of Questions

Following past scholarship (Sipocz et al., 2021), the coding rubric was designed using both deductive and inductive modes of reasoning (Armat et al., 2018). Analyses led by a directed or deductive approach begin with the identification of an initial set of codes based on prior literature (Armat et al., 2018). Meanwhile, in inductive content analyses, codes are derived directly from the data (Armat et al., 2018). We employed both deductive and inductive approaches to ensure that certain pertinent assumptions informed the analysis although also mindful that new categories would emerge inductively.

We started by identifying a set of categories based on prior research to develop a preliminary codebook. The analysis was subsequently conducted in several stages, with each question read twice by the authors to ensure familiarity with the data. The aim of the first reading was to ascertain the validity of the initial set of categories, as well as to modify the codebook until all variables were refined and defined clearly. During this first reading, a new category was added whenever a question featured a particular attribute that could not be suitably coded into any of the existing categories, and appeared frequently enough to warrant its own category. The aim of the second reading was to make sure we had a framework sufficiently representative of the different types of questions so as to finalize the coding rubric.

To ensure rigor in the analysis, the two coders had regular discussions during which any discrepancies were reviewed and adjudicated. Areas of major overlap were identified and sectioned into broader themes. The percentage agreement between the two raters was 95.5% with a weighted Cohen’s kappa of 0.91 (*p* < .001), showing high inter-rater reliability. Five themes emerged from this iterative procedure. It must be highlighted that a question may be put under more than one theme. As mentioned in earlier work, categories in a content analysis need not be mutually exclusive although they should be internally homogenous (i.e., coherent within themes) and externally heterogenous (i.e., distinct from each other) as far as possible (Bengtsson, 2016; Sipocz et al., 2021; Tesch, 1990).

## Results

### Summary of Insights From Content Analysis of Questions on Quora

**Figure 2.**
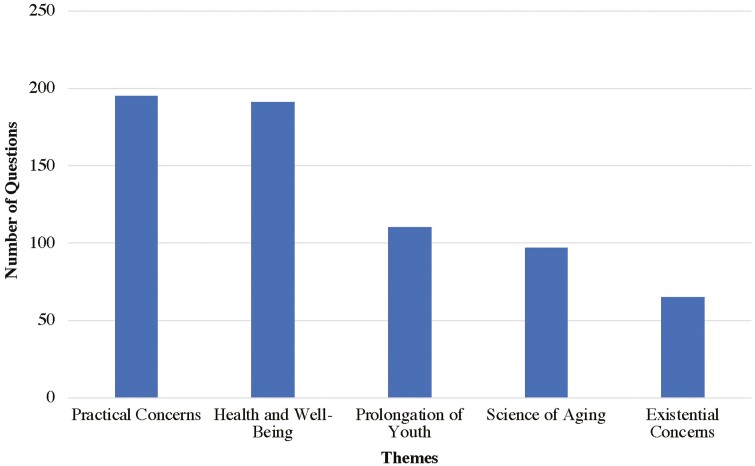
Themes of questions about later life posted by Quora users.

We identified five themes through a content analysis of questions about later life. The biggest theme (30%; *N* = 195) dealt with ‘Practical Concerns’ (Theme 1). The next biggest theme (29%; *N* = 191) was about “Health and Well-Being” (Theme 2). Questions parked under Theme 3 were about the ‘Prolongation of Youth’ (16%; *N* = 110) and those under Theme 4 were about ‘Science of Aging’ (15%; *N* = 97). ‘Existential Concerns’ were classified under Theme 5 (10%; *N* = 65). See Figure 2 for a summary.

### Practical Concerns (Theme 1; 30%)

Under this theme, users asked a diverse range of questions related to retirement planning, financial planning, and living arrangements.

Many expressed concerns about the timing of retirement preparations, with questions such as “At what point in someone’s life should they start to make preparations for retirement?” and “Is $500k enough to retire at the age of 60?” Users solicited advice on retirement planning, asking, “What are some things that people wish they had known sooner about aging and retirement planning?” Some also asked for suggestions for “hobbies and interests” that older adults can pursue to “ward off boredom.”

Financial concerns were a common topic. Users questioned the “best time to take [one’s] social security” and wanted to know how “Social Security protect[s] seniors from economic insecurity in retirement.”

Living arrangements were another area of interest. One inquirer sought clarification on “the differences between retirement homes, nursing homes and assisted living facilities” as well as the “factors to consider when choosing among these options.” Safety concerns were also raised, with people asking, “Are tubs with low walls safer for older people than those with higher sides?” and “What are some simple things that can be done to make sure that one’s home is aging-proofed and safe?”

### Health and Well-Being (Theme 2; 29%)

Questions belonging to Theme 2 spanned a whole spectrum of health-related concerns about aging. Many users were interested to learn about practical ways to maintain their health and well-being.

Most questions were about preventive measures to “ensure a longer and healthier life.” One asked, “How can senior citizens stay mentally active and engaged despite physical constraints due to age-related health issues?” Another inquired, “What physiological changes occur as a result of physical and cognitive aging, and how can they be managed most effectively?” Many were curious about whether there were specific “exercises,” “foods” or “supplements” that could facilitate healthy aging.

Some were interested about whether the aging body could still achieve physical goals such as “build muscle and strength” or “run a marathon.” There were questions about how to cope with or prevent specific health conditions such as “knee pain,” “incontinence,” “hearing loss” and “dementia.”

In addition to concerns about physical and cognitive health, posters also flagged queries about mental and social well-being. There were some who wondered if “depression and loneliness” were “normal” among older persons. A small fraction of posts honed in on the well-being of older persons who are childless, single, or without family. For example, a self-proclaimed “childless widow” asked fellow users if she was “destined for an unavoidable[,] lonely old age.” One thought about whether “having children guarantee[d] anything in old age” and another asked how those who are “single” or “unmarried” “go through old age” and “cope with loneliness.”

### Prolongation of Youth (Theme 3; 16%)

Posts under this theme revolved around ways to prolong youth and reduce visible signs of aging. Individuals had an assortment of questions, from the safety and effectiveness of antiaging treatments to advice on how to prevent premature aging of the skin, including the use of skincare products, lifestyle modifications, and natural remedies.

Some of the inquiries pertained to whether “excessive exposure to sunlight” or “cold weather” can age the skin prematurely, and if “glycolic acid” can reduce “wrinkles and fine lines.” Others hankered after “the secret to having youthful skin forever.” A few brought up questions regarding “noninvasive anti-aging treatments.”

A group of users probed into “ways to slow down the aging process and extend natural life spans beyond what is currently thought to be possible.” Some asked about whether “certain hormonal or gene therapy treatments slow down the aging process” and whether “anti-aging supplements” can “reverse aging.” The safety, effectiveness, and affordability of anti-aging treatments were matters of concern for select individuals.

There were questions that hinted at the phenomenon of gendered ageism. One pondered how “aging women come to grips with [the] loss of attractiveness” and how older women feel looking at pictures of their former, “more attractive” selves.

### Science of Aging (Theme 4; 15%)

Queries filed under Theme 4 covered a plethora of topics related to the scientific underpinnings of aging. In these queries, users showcased a desire to comprehend the mechanisms driving the cellular, biological, and psychological changes that occur as people grow older.

Some of these questions investigated the link between aging and specific health matters, such as memory loss, changes in skin condition, or sleep patterns. For example, one asked, “How does sleep change as we age, and what can we do to ensure we’re getting enough restful sleep as we get older?” Another was uncertain about whether older people confronted “a higher chance of getting lung, colon, or breast cancers than younger people.” There were also questions about why older people “lose strength in their legs” and how one’s “sense of smell” changes over time.

Several posts dove into the psychological and emotional changes that accompany the aging process. For instance, one query was about the “connection between age and wisdom.” A user wanted to know if it was true that “older people have less emotion than younger people.” An inquiry was made about how “one’s level of optimism” shifts over time.

There were questions about the genetics of aging and cellular changes that take place when growing older. Someone asked, “What are telomeres and how can lengthening them impact the aging process?” Another deliberated on the “root cause of aging.” One question was about how the “composition of gut microbiome” changes with age.

### Existential Concerns (Theme 5; 10%)

This theme dealt with the psychological and emotional aspects of aging, including the fear of death, feelings of regret, loss of purpose, and changes in perspectives on life.

Some posed questions about the “fear of death” and whether “people who live to old age [are] truly ready to die when their time comes.” Others inquired about strategies for coming to terms with one’s mortality, with one asking, “How do older people cope with the feeling that no matter what they do, they have very few years to live?”

This theme also touched on how growing older may impede the fulfillment of personal goals. One person mused, “Has any of your goals, dreams or desires been affected by getting older?”

Regret was another recurring theme. For example, one inquirer was eager to know how regret “affect[s] people after they reach old age.” Another wondered, “What can people do to avoid regret at an older age?”

Finally, a few questions explored the complex relationship between growing older and feeling contented, such as “Do most older people wish they were younger and/or could go back in time, or do you feel content with your age and where you are?”

## Discussion

This study is significant in being the first to look at the types of questions individuals have regarding later life using a question-and-answer website. Our content analysis of questions posted on Quora between 2010 and 2023 unveiled five themes that collectively encapsulate the social and cultural zeitgeist of the past decade in relation to aging: “Practical Concerns” (Theme 1), “Health and Well-Being” (Theme 2), “Prolongation of Youth” (Theme 3), “Science of Aging” (Theme 4), and “Existential Concerns” (Theme 5).

Practical considerations took precedence in the minds of many users. As people contemplate their own or their parents’ retirement, worries about how to find purpose and fulfillment in later life usually become more prominent. Research has evinced that it is not uncommon for people to feel apprehensive about their retirement years. Some even hold off retirement precisely because they foresee struggling to navigate the exit from their roles at work (Bordia et al., 2020). This was evident in questions where users sought tips on how to stave off boredom in retirement. Users also had questions about the more tangible and material aspects of later life such as finances and living arrangements. Planning for retirement is associated with improved well-being and satisfaction during retirement (Noone et al., 2009), though only a sliver of the populace actually engages in such planning (Hurwitz and Mitchell, 2022; Millan and Thayer, 2022). In fact, many older persons have expressed regret over past decisions that rendered them susceptible to financial insecurity in old age (Hurwitz and Mitchell, 2022; Millan and Thayer, 2022). The emphasis on practical considerations among Quora users suggests that there may be value in developing programs that better prepare the public for life in retirement.

Concerns related to health and well-being in old age attracted significant interest among Quora users, which is indicative of the importance placed on maintaining good health as people age. This comes as no surprise considering that health constitutes a fundamental concern of human existence. The interest in such issues implies that users are not only interested in prolonging their life span, but also their health span, meaning the period in which they are free from disease (Hansen and Kennedy, 2016). Good health in later life is essential for overall well-being and helps older adults maintain their independence, mobility, and ability to engage in activities they enjoy (Abud et al., 2022). However, since aging has long been synonymous with declining health (Lindland et al., 2015; Sweetland et al., 2017), some may have posed such queries to feel a greater sense of control over their health, or to cope with the anxiety about the physical or cognitive changes that come with growing older. When left unchecked, this anxiety can affect one’s quality of life (Yawar et al., 2022).

The numerous questions about the use of antiaging interventions signal a preoccupation with youthfulness among the Quora community. To a certain extent, these questions reveal a sense of anxiety about aging, with many anticipating that growing older would be detrimental to one’s physical appearance. This could be attributed to the sociocultural idealization of youth and the proliferation of antiaging paraphernalia, both of which reinforce the idea that youthfulness is a prerequisite for social desirability (Clarke and Griffin, 2008). Although the use of antiaging products is associated with certain psychological benefits (McKeown, 2021; Sadick, 2008), many gerontologists decry the antiaging industry for stigmatizing a natural transition in life (Flatt et al., 2013). The prevalence of these questions signifies that for some, old age should be avoided and delayed rather than accepted. When internalized, such negative views of old age may worsen health outcomes among older persons (B. Levy, 2009a). Thus, there is a need to manage the underlying fears that fuel the desire to prolong youthfulness.

A number of users had questions about the mechanisms responsible for the biological, psychological, and mental changes that occur as one ages. Despite being a universal experience, aging is a process often shrouded in confusion. At present, research on the public understanding of aging tends to dwell more on stereotypes about old age (Jimenez‐Sotomayor et al., 2020; Sipocz et al., 2021) rather than the scientific principles underlying the aging process. Improving scientific literacy on aging among the public may help individuals maintain their health as well as eliminate misconceptions about aging.

For a certain segment of the Quora population, it was not so much aging that was the primary source of unease as it was the notion that aging is a harbinger of death. Several individuals also vocalized concerns about not having lived life to the fullest. Developed by Greenberg and colleagues (1986), the terror management theory proposes that the human urge to survive, combined with the awareness of the looming threat and inevitability of death, induces a persistent feeling of anxiety in the human psyche. Although anxiety about aging and anxiety about death are distinct concepts (Lasher and Faulkender, 1993), the two are obviously interconnected. Anxiety about aging is often induced by anxiety about death (Benton et al., 2007), which is a significant predictor of psychopathology (Menzies et al., 2019).

### Implications

The findings from this study have various implications. First, since many users have questions or concerns about retirement, it would be prudent to implement programs that encourage individuals, regardless of age, to plan for later life. This is especially important since negative attitudes toward growing older may stem from worries about retirement, a phase many dread (Bordia et al., 2020). Companies could invest in programs that educate employees on the significance of planning for later life, such as seminars or programs on financial literacy, long-term care planning, housing counseling, volunteering, continuing education, and wellness. The incorporation of such initiatives into the workplace could normalize retirement planning, which will eventually benefit employees as they transition out of the workforce.

Second, in view of the public’s interest in learning more about how to age healthily, practical steps could be taken to improve knowledge on the subject. Organizing workshops and seminars targeted at older adults may equip them with knowledge on how to stay healthy and active as they age, which could also alleviate fears and anxieties surrounding the aging process (Lasher and Faulkender, 1993). In addition, integrating content on healthy aging into school curricula may inculcate more positive attitudes toward aging among younger generations. For instance, individuals could be taught that regular exercise, well-planned diets, and mental wellness are essential components of healthy aging. Furthermore, public health campaigns could be set up to promote healthy aging across all age groups and to raise awareness of the impact of healthy aging on one’s well-being. Leveraging the reach of popular media, be it through television broadcasts, commercials, or various social media platforms, may also be worthwhile. For example, advertisements could feature tips for healthy aging or showcase older adults proactively engaging in health-promoting behaviors.

Third, it is essential to facilitate conversations about advanced care planning in clinical care settings. Healthcare professionals in this domain must be equipped with the necessary skills and resources to foster a culture of patient-centered care that promotes the autonomy and dignity of individuals. This will empower patients and their families as they traverse the complex landscape of aging-related healthcare.

Fourth, there is a pressing need to curate resources tailored specifically for those who find themselves having to juggle the responsibilities of supporting their aging parents and bringing up their children. Key stakeholders like employers, community organizations, and healthcare institutions, play a pivotal role in supporting them as they navigate this balancing act.

Fifth, notwithstanding the psychological benefits linked to employing antiaging interventions such as greater well-being and reduced appearance-related distress (McKeown, 2021; Sadick, 2008), it is vital to nurture a culture that does not equate old age with unattractiveness. Besides improving the self-esteem of individuals entering their advanced years (Sabik, 2015), this will also ease the layperson’s fear of growing older. One area where this cultural shift could be implemented is in the beauty industry. Even though beauty conglomerates are distancing themselves from the term “anti-aging,” many have opted for euphemisms such as “regeneration,” “renewal,” “vitality” that continue to betray a fixation with a youthful countenance (Hess, 2017). To achieve true inclusivity, certain products could be marketed as meant for the nourishment of older skin (Walsh, 2017). Wrinkles could also be normalized and even celebrated in later life through advertisements.

Sixth, since aging is a process that everyone is bound to undergo, it only makes sense that the science behind it is demystified. One way to do so is to enhance public outreach efforts by scientists and researchers. This could involve communicating complex scientific concepts in a more accessible way to the public, such as by using plain language and visual aids. Additionally, science education programs in schools and universities could cover more topics related to aging. Promoting scientific literacy about aging may help individuals make sense of their aging bodies when they eventually reach later life, as well as dispel myths about growing older.

Finally, as disentangling old age from death is not entirely viable, normalizing conversations about the finitude of life may promote a greater acceptance of death (Chu, 2019), which could in turn reduce the negative associations it has with aging. To this end, it may be valuable to create more opportunities for people to engage in discussions about death and dying, for instance through community events, support groups or educational programs. By teaching individuals to embrace death as an inherent part of existence, individuals may develop a healthier perspective on aging that is not dominated by the fear of death. This could then allay anxiety about both aging and death (Benton et al., 2007).

### Limitations and Future Directions

There are several limitations to this study. First, as demographic information is not provided by most Quora users, we could not explore how questions about old age differed across variables such as age, gender, or socioeconomic status. Furthermore, Quora does not publish information about the gender and ethnic make-up of its user base. A future study could examine how different demographic factors inform the kinds of concerns individuals have about aging. Second, over half of Quora’s users are aged between 18 and 34 years old (Ruby, 2023), which means they are not representative of the wider public. Nonetheless, these questions reflect the kinds of concerns or questions individuals have about their own aging journeys or those of their older relatives. Third, we were unable to determine the proportion of the questions originating from different countries. However, Quora’s biggest clientele is the United States where there are around 140 million users, followed closely by India where there are roughly 100 million users (Agarwal, 2023). This affects the generalizability of our findings. Future research could investigate the influence of cultural factors on concerns about aging. Fourth, although we picked topics with a sizable following, we may have overlooked other relevant topics. Fifth, our analysis only focused on the questions asked by users on Quora and did not consider their corresponding answers. An examination of the responses to these questions would offer insight into the depth of public knowledge regarding aging. Another avenue for future inquiry is a longitudinal study that tracks changes in concerns about aging across the life span. Other methodologies such as surveys (Steglitz et al., 2012) or big data analytics could also be employed to identify the public’s concerns about later life.

## Conclusion

In conclusion, this study adds a new dimension to existing scholarship regarding public opinion on aging. Our results suggest that there is a need to address questions that the public has about aging, particularly those involving practical issues and health. Growing old is unavoidable and with the population aging at a rapid pace, assuaging such concerns is of paramount importance. By doing so, individuals can approach the aging process with greater clarity and an elevated sense of empowerment.

## Data Availability

All data analyzed in this study are publicly available at www.quora.com. This study was not preregistered.
